# RNA Editing Responses to Oxidative Stress between a Wild Abortive Type Male-Sterile Line and Its Maintainer Line

**DOI:** 10.3389/fpls.2017.02023

**Published:** 2017-11-28

**Authors:** Jie Xiong, Tao Tao, Zhi Luo, Shuaigang Yan, Yi Liu, Xinqiao Yu, Guolan Liu, Hui Xia, Lijun Luo

**Affiliations:** ^1^College of Plant Sciences and Technology, Huazhong Agricultural University, Wuhan, China; ^2^Shanghai Agrobiological Gene Center, Shanghai, China

**Keywords:** pentatricopeptide repeat protein, PPR, stress acclimation, stress tolerance, transmembrane domains, *ccmB*, *ccmC*

## Abstract

RNA editing of mitochondrial gene transcripts plays a central role during plant development and evolutionary adaptation. RNA editing has previously been reported to differ between the rice cytoplasmic male sterile (CMS) line and its maintainer line, which has been suggested as a cause for their different performances under environmental stress. To specifically test this hypothesis, a wild abortive (WA) CMS line (Huhan-1A) and its maintainer line (Huhan-1B) were utilized to investigate performances in response to oxidative stress, as well as RNA editing efficiencies on transcripts of six selected mitochondrial genes. Compared to the maintainer line, Huhan-1A represented both lower plant height and total antioxidant capacity, possessed higher total soluble protein and chlorophyll contents, accumulated less H_2_O_2_ content on the 3rd day after treatment (DAT), and exhibited higher survival ratio after re-watering. Furthermore, a total of 90 editing sites were detected on transcripts of six mitochondrial genes (*atp9, nad2, nad7, nad9, ccmB*, and *ccmC*) in both Huhan-1A and Huhan-1B on the 0, 1st, and 3rd DAT. Forty-eight sites were furthermore determined as stress-responsive sites (SRS). Generally, in response to oxidative stress, SRS in Huhan-1A increased the resulting editing efficiencies, while SRS in Huhan-1B decreased the resulting editing efficiencies. In addition, 33 and 22 sites at *ccmB* and *ccmC* were differentially edited between Huhan-1A and Huhan-1B, respectively, on the 0, 1st, and 3rd DAT. Editing efficiencies of *ccmB* and *ccmC* were generally lower in Huhan-1A (*ccmB*, 37.3–47.8%; *ccmC*, 41.2–52.3%) than those in Huhan-1B (*ccmB*, 82.6–86.5%; *ccmC*, 81.0–82.9%). Deficiencies of RNA editing in Huhan-1A at *ccmB* and *ccmC* could lead to the loss of transmembrane domains in their protein structures. Consequently, differences in RNA editing at *ccmB* and *ccmC* between the WA-CMS line and its maintainer line partially explained their different performances under stress. Moreover, we detected differences in expressions of pentatricopeptide repeat (PPR) genes between both lines, as well as significant correlations with RNA editing. Our study indicated potential associations of RNA editing and PPR genes in rice tolerance to abiotic stresses. However, the underlying molecular mechanisms of stress-adaptation, which are attributed to RNA editing on transcripts of mitochondrial genes, require further investigation.

## Introduction

RNA editing is a post-transcriptional mechanism that alters the nucleotide sequence of an RNA molecule after its transcription (Hammani and Giege, 2014). This editing of mRNAs often leads to a change of identity of an amino acid that has been encoded by the edited gene. In plants, RNA editing typically occurs as C-to-U conversions at the first or second positions of an amino acid codon (Fujii and Small, 2011; Hammani and Giege, 2014). Since the first pentatricopeptide repeat (PPR) protein was identified and functionally described, PPR proteins have been proven to play a central role in the process of RNA editing (Hammani and Giege, 2014). PPR proteins have been reported to contain conserved domains to edit mRNA of organelle genes and have formed an extended family during plant evolution (Hammani and Giege, 2014). More than 400 PPR genes have been predicted in rice (O'toole et al., 2008; Fujii and Small, 2011).

RNA editing has various biological functions (Fujii and Small, 2011; Hammani and Giege, 2014). It can introduce the translation initiation codon AUG from an ACG codon for several genes (Kadowaki et al., 1995) and can promote RNA splicing by affecting the intron structures (Castandet et al., 2010; Farré et al., 2012). The alteration of editing at a specific site of a mitochondrial gene can harmfully impact plant growth, development, fertility, and seed development (Kim et al., 2009; Toda et al., 2012; Liu et al., 2013; Yap et al., 2015). In addition, RNA editing has its significance during evolution (Fujii and Small, 2011) and has been suggested to play a role in plant adaptation to land conditions (e.g., extreme temperatures, UV, and oxidative stress) when plants colonized the land (Fujii and Small, 2011; Hammani and Giege, 2014). Accordingly, numerous studies have reported RNA editing on transcripts of chloroplast and suggested mitochondrial genes to be responsive to various environmental stressors: e.g., rice to the cold (Kurihara-Yonemoto and Kubo, 2010) and maize to the heat (Nakajima and Mulligan, 2001). Many PPR gene mutants can further alter their morphological appearances under stress conditions compared to the wild-type (Tan et al., 2014; Zhu et al., 2014). Therefore, RNA editing in likely playing a role in plant resistance to a given environmental stress and it is necessary to explore how this influences plant responses to environmental stresses.

Cytoplasmic male sterility (CMS) describes the maternally inherited inability to produce functional pollen in plants. The successful hybrid rice breeding in China is largely attributed to the utilization of CMS resources and their comparatively high rates of out-crossing (Cheng et al., 2004, 2007). CMS always originated via chimeric open reading frames as a result of rearrangements of mitochondrial genomes (Hanson and Bentolila, 2004; Luo et al., 2013; Wang et al., 2013). Among various CMS resources, the wild abortive CMS (WA-CMS) system is widely used in breeding, accounting for ~99% of all hybrid rice cultivars (Cheng et al., 2007; Luo et al., 2013). Recently, the Honglian type CMS rice line (HL-CMS) has been reported to be more sensitive to oxidative stress than its maintainer line due to its greater inhibition of growth under stress (Hu et al., 2010; Li et al., 2012; Wang et al., 2013). The male sterile gene *ORF79* has been suggested as the cause of this observed sensitivity (Li et al., 2012). However, the performances of WA-CMS and its maintainer line in response to stressed environments have not been evaluated. If they responded differently to environmental stresses, the underlying causes could be different from HL-CMS as the transcript of the male sterile gene *WA352* does not accumulate in leaves (Luo et al., 2013). Interestingly, a specific WA-CMS rice line has been reported to possess differences in RNA editing of many mitochondrial genes, compared to its maintainer line under normal conditions (Hu et al., 2013). As differences in RNA editing at mitochondrial genes could result in different morphological appearance (Kim et al., 2009; Toda et al., 2012; Liu et al., 2013; Yap et al., 2015), this could provide a reasonable explanation for potential differences in performance between WA-CMS and its maintainer line under a given stress. However, any differences in responses of RNA editing to a particular stress between WA-CMS and its maintainer line have not been fully studied to date. WA-CMS and its maintainer line provide good opportunities to learn more about the role of RNA editing on transcripts of mitochondrial genes in response to environmental stress.

Oxidative stress commonly occurs in combination with various abiotic stresses, such as drought, heat, cold, and heavy metal stress (Millar et al., 2011; Petrov et al., 2015; Saxena et al., 2016). Enhancing the tolerance of a plant to oxidative stress can help to improve its tolerances to various other abiotic stresses (Gill and Tuteja, 2010; Hossain et al., 2015). For this study, a WA-CMS line (Huhan-1A) and its maintainer line (Huhan-1B) were utilized to investigate both their respective performances and efficiencies of RNA editing under oxidative stress. Huhan-1A has been modified from a typical WA-CMS line (Zhenshan-97A) via introducing water-saving and drought-resistant (WDR) characteristics from an upland rice accession (Luo, 2010). Huhan-1A shows a considerable level of drought-resistance in the field and has therefore been widely used for WDR breeding (Luo, 2010). By comparing the differences of RNA editing between Huhan-1A and Huhan-1B, we addressed the following questions: (1) Will there be differences in performance between the CMS and its maintainer line under oxidative stress? (2) Will RNA editing on transcripts of mitochondrial genes differ between the CMS and its maintainer line in response to oxidative stress? (3) If it were true, what would be the potential cause? (4) Can differences in RNA editing between CMS and its maintainer line explain differences in performance under oxidative stress?

## Materials and methods

### Plant materials

A WA-CMS line (Huhan-1A) and its maintainer line (Huhan-1B) were used for our study. Huhan-1B has been developed from an upland rice line (EAIC139-55-1-23) by the International Rice Research Institute (IRRI) via selfing and purifying. Huhan-1A was developed via backcrossing between Zhanshan97A (a typical WA-CMS rice line) × Huhan-1B for at least seven generations, during which Huhan-1B was used as recurrent pollen donor (Luo, 2010). Huhan-1A and Huhan-1B have extremely high similarities (>99.9%) in their nuclear genomes (Table S1). However, hundreds of PPR genes were detected to have SNP and/or InDel between Huhan-1A and Huhan-1B (unpublished data). Huhan-1A/Huhan-1B contains a substantial drought-tolerance level and has therefore been used to breed numerous hybrid water-saving and drought-resistance rice varieties (Luo, 2010). As a result, it has been granted the national certification of a rice variety in 2007 (CNA20040206.4). As both are tolerant varieties, any alteration in the sensitivity to oxidative stress in Huhan-1A could easily be detected via comparison with Huhan-1B.

### Rice cultivation and experimental treatment

Seedlings of WA-CMS and its maintainer line were transplanted to a 96-well plate 3 days after germination and cultivated in a nutrient solution (Xia et al., 2017). They were placed in a growth chamber (14 h of light at 30°C and 10 h of darkness at 20°C with 70% relative humidity). Each rice line had eight plates with 48 individuals per plate. After 7 days of growth in normal nutrient solution, four plates (replicates) of each rice line were treated with a nutrient solution with 60 mmol/L H_2_O_2_ in the final concentration. A further four replicates were kept in normal solution as controls (CK). Normal and H_2_O_2_-added nutrient solutions were renewed once every 3 days.

### Sampling strategy and measurements for physiological traits

The above ground parts (including both stem and leaf tissue) of three individual seedlings per plate were mixed sampled used to measure stress-related physiological traits on 0 (before treatment), 1st, and 3rd day after treatment (DAT) for the oxidative-stressed plates. Each treatment had four biological repeats (four plates). Six oxidative stresses related to physiological traits were measured, including the total soluble protein (TSP) content, the total antioxidant capacity (AOC), the H_2_O_2_ content, the specific activity of glutathione peroxidase (GPX), the specific activity of superoxide dismutase (SOD), and the specific activity of catalase (CAT). GPX activity was measured with the Glutathione Peroxidase Cellular Activity Assay Kit (cat. no. CGP1-1KT, Sigma). TSP (Product# A045-2), AOC (Product# A015), H_2_O_2_ content (Product# A064), SOD activity (Product# A001-1), and CAT activity (Product# A007-1) were measured following the manufacturer's instructions of their corresponding test kits from Nanjing JianCheng Bioengineering Institute, China (http://elder.njjcbio.com/html_en/search.php). Since the degradation of chlorophyll is one of the most noticeable physiological responses of rice to oxidative stress, well-maintained chlorophyll levels could be a key parameter to describe the tolerance to oxidative stress. Consequently, chlorophyll was ethanol-extracted from leaf tissues and quantified *via* spectrophotometry. Briefly, 0.05 g fresh leaves from seedlings were crushed and put into 20 ml 95% ethanol for 24 h in the dark. The 250 ul supernatant was then measured at both 649 nm and 665 nm. The content of chlorophyll-a (Ca) (mg/L) was calculated via the empirical formula: 13.95^*^A665–6.88^*^A649. The content of chlorophyll-b (Cb) (mg/L) was calculated via the empirical formula: *Cb* = 24.96^*^A649-7.32^*^A665. The chlorophyll content was calculated as (Ca + Cb) and its unit was converted to mg/g of fresh leaf.

To evaluate rice growth during the stress, plant height was measured from two individual seedlings per plate for both CK and oxidative-stressed plates on the 0, 1st, and 3rd DAT using four biological repeats (four plates). These stress-exposed plates were re-cultivated in normal nutrient solution on the 5th DAT and the survival ratio was measured 2 days later from the remaining seedlings (~24 seedlings per plate). Furthermore, another three individual seedlings were mixed sampled per plate on the 0, 1st, and 3rd DAT to extract their RNA for testing RNA editing and gene expression, using three biological repeats (three plates). The last plate was used to extract DNA for gene cloning. All seedling samples were immediately frozen in liquid nitrogen and kept at −80°C until further use.

### Genes selected for sequencing to detect RNA editing

As previously reported, dysfunction of the mitochondrial electron transport chain (ETC) could lead to ROS over-production (Keunen et al., 2011) and impact the plant stress-adaptation (Gleason et al., 2011). Therefore, six mitochondrial genes (*atp9, nad2, nad7, nad9, ccmB*, and *ccmC*) related to ETC were selected to test RNA editing on their transcripts. *atp9* encodes the ATP synthase subunit nine. It is an indispensable part of the electron transport chain of respiration. Efficiencies of RNA editing at sites of *atp9* have already been determined previously in a different WA-CMS line (Zhengshan97A) and its maintainer line (Hu et al., 2013). This could be used as reference to validate the reliability of our method. The mitochondrial genes *nad2, nad7*, and *nad9* encode subunits of NADH dehydrogenase complex I in the mitochondrial respiratory chain. Deficiencies of RNA editing within these genes can negatively impact both growth and development (Toda et al., 2012). *ccmC* and *ccmB* have been suggested to be stress-resistance genes in upland cotton (*Gossypium hirsutum* L.) (Zhang et al., 2015). Although the functions of *ccmB* and *ccmC* in rice have not been fully understood so far, both genes are highly conserved between rice and upland cotton with 94 and 93% identitical protein sequences, respectively. Moreover, they have also been reported to be associated with the maturation of Cytochrome c in maize *emp9* mutants, leading to dysfunction of ETC (Yang et al., 2017). Since Huhan-1A and Huhan-1B may potentially have differences in tolerance to oxidative stress, *ccmB* and *ccmC* were selected as target genes for testing RNA editing between both rice lines during stress exposure. Finally, all these mitochondrial genes contain a considerable number of editing sites that have been annotated by the National Center for Biotechnology Information to increase the detection efficiency for RNA editing (Figure S1).

### DNA extraction and cloning of mitochondrial genes

Samples from one stressed plate were used to extract DNA to amplify target regions of six selected mitochondrial genes in Huhan-1A and Huhan-1B, via their corresponding primers (Table S2). Target bands of the PCR product of each mitochondrial gene were recycled and sequenced after cloned to the pEASYR-Blunt Simple Cloning Vector (Product#CB111, TRANSGEN BIOTECH Company, China). DNA sequences of the amplified regions were then compared between Huhan-1A and Huhan-1B to determine homogeneities at the editing sites (Figure S1).

### RNA extraction and testing of RNA editing efficiency

Samples of all other three stressed plates were used to extract RNA to test both RNA editing and gene expressions. Total RNA was extracted via the TRNzol-A+ Total RNA Reagent (TIANGEN, Beijing, China), using entire seedlings (containing both stems and leaf tissues) that were harvested on the 0, 1st, and 3rd DAT, respectively. cDNA was obtained via reverse transcription of total RNA using the PrimeScript® RT reagent Kit (Takara Biotechnology, Dalian, China) and following the manufacturer's instructions. The target regions of these selected mitochondrial genes were amplified, using corresponding primers (Table S2). The target-amplified band of each mitochondrial gene was recycled from the PCR product retrieved from the agarose gel. Target amplified bands of six genes of the same plate were mixed and sent for sequencing *via* Illumina HiSeq 2500 (Biomarker Technologies Co, LTD, Beijing, China) with at least 10 M of clean data per sample. Three replicates were utilized for the RNA editing testing. Quality-controlled reads were mapped to the reference gene sequences of Huhan-1A and Huhan-1B that originated from the DNA sequencing above (Figure S1). Since RNA editing can lead to C–T (or G–A) alterations in the cDNA, these types of mismatched bases were allowed during mapping reads to the reference gene sequence. The RNA editing efficiency at one site was expressed as the proportion between edited transcripts and total transcripts. If a site of a transcript was edited, the C/G base (wild type) should be altered to the T/A base (edited type). Since one editing site could be detected hundreds of times *via* sequencing, the number of wild type (C/G) or edited type (T/A) of bases could then be counted at this particular site. The editing efficiency at one site could then be calculated as: counts of edited bases (T and A)/total counts of bases ^*^ 100%. The code of an editing site was named by its position after the first base of the translation start site.

### Expression quantification for selected mitochondrial genes and three PPR genes *via* qPCR

Abundance of transcripts of the mitochondrial gene and expression of PPR genes may both potentially impact editing efficiency, as these transcripts formed the substrate of PPR genes for RNA editing. To test this, we investigated the expressions of six mitochondrial genes and three PPR genes (*ogr1, emp5*, and *mpr25*) in response to the oxidative stress of Huhan-1A and Huhan-1B and conducted correlation analyses between their respective expressions and RNA editing efficiencies. These three PPR genes have been reported to play essential roles in RNA editing of mitochondrial genes in rice (Kim et al., 2009; Toda et al., 2012; Liu et al., 2013; Yap et al., 2015), including *nad9* and *ccmC* (Kim et al., 2009; Liu et al., 2013). In addition, both *ogr1* (LOC_Os12g17080) and *emp5* (LOC_Os01g72930) differed in their sequences between Huhan-1A and Huhan-1B, while *mpr25* (LOC_Os04g51350) did not (unpublished data). Real-time PCR was conducted using Hard-Shell® 96-Well PCR Plates (BIO-RAD, USA), utilizing the CFX96TM Real-Time System (BIO-RAD, USA). The utilized reaction system contained 10 μl of 2x SYBR Premix Ex TaqTM (Takara Biotechnology, Dalian, China), 20 ng cDNA, and 0.1 μM of gene-specific primers (Table S2) at a final volume of 20 μl. The used thermal cycle was 95°C for 30 s, followed by 40 cycles at 95°C for 5 s, and 60°C for 31 s, using an additional dissociation stage. Each gene used three biological replicates, each of which was subjected to three technical qPCR replicates. The housekeeping gene *Actin2* was used as a reference to calculate relative expression levels of each gene.

### Data analysis

Independent *t*-tests were conducted to test for any differences in measured physiological traits, RNA editing efficiencies, and gene expressions between both Huhan-1A and Huhan-1B and at each sampling time point (for the 0, 1st, and 3rd DAT). One-way ANOVA (SNK method) was conducted to detect differences in the measured physiological traits, RNA editing efficiencies, as well as gene expressions among the 0, 1st, and 3rd DAT, respectively, in Huhan-1A and Huhan-1B. Since one editing site may have contributed in either a minor or major way to the normal function of a particular mitochondrial protein, we could not estimate the significance of each editing site for a mitochondrial gene. We therefore calculated a general editing efficiency, which enabled us to describe a general tendency (increasing or decreasing) of RNA editing in response to oxidative stress (one-way ANOVA). Furthermore, we detected general differences in RNA editing between Huhan-1A and Huhan-1B (via paired *t*-test) for each mitochondrial gene. This was calculated as the means of editing efficiencies at all editing sites of a particular mitochondrial gene, and represented general differences either in RNA editing between Huhan-1A and Huhan-1B, or among time points. In addition, general differences in editing efficiencies at stress-responsive sites (SRS) between Huhan-1A and Huhan-1B were calculated as the means of differences of editing efficiencies of all SRSs between Huhan-1B and Huhan-1A. Paired *t*-test conducted to test for any differences among the 0, 1st, and 3rd DAT. Correlation analyses were conducted between gene expressions and editing efficiencies to test for any impacts caused by mitochondrial and PPR genes on RNA editing. Since the general editing efficiency was low for Huhan-1A and high for Huhan-1B, we defined the fully edited forms of *ccmB* and *ccmC* as Huhan-1B type, while defining non-edited forms as Huhan-1A type. Transmembrane helices in ccmB and ccmC proteins of fully edited (Huhan-1B type) or non-edited (Huhan-1A type) forms were predicted via TMHMM (http://www.cbs.dtu.dk/services/TMHMM/) using default settings (Krogh et al., 2001).

## Result

### Morphological and physiological responses of rice seedlings to oxidative stress in Huhan-1A and Huhan-1B

**Figure 1:**
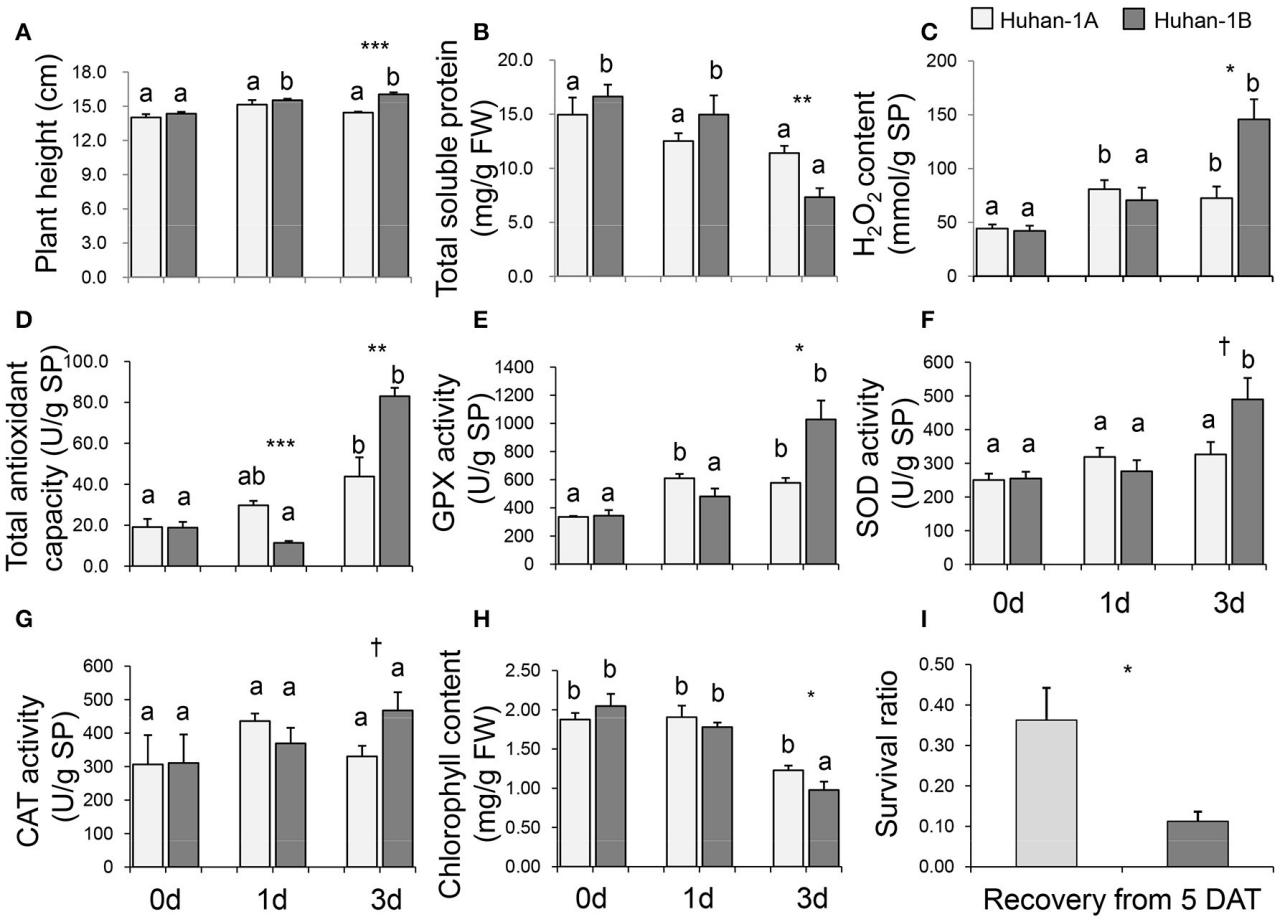
Physiological traits (**A**, plant height; **B**, total soluble protein; **C**, H_2_O_2_ content; **D**, total antioxidant capacity; **E**, GPX activity; **F**, SOD activity; **G**, CAT activity; **H**, chlorophyll content; **I**, survival ratio) measured in both Huhan-1A and Huhan-1B under oxidative stress. ^*,**,***,†^Indicates significant difference at *p* < 0.05, *p* < 0.01, *p* < 0.001, and *p* < 0.1 between Huhan-1A and Huhan-1B via independent *t*-test at one time point (*n* = 4). Different letters indicate significant differences (*p* < 0.05) among time points in Huhan-1A or Huhan-1B based on one-way ANOVA (SNK method).

Huhan-1A and Huhan-1B had very similar plant heights in the CK condition (see Figure 1A). The growth of both Huhan-1A (Figure S2A) and Huhan-1B (Figure S2B) was greatly inhibited under oxidative stress since their plant heights of the stressed condition were significantly lower than those of the CK condition. However, Huhan-1B showed a slight but significant increase in its plant height during the stress, which led to significantly higher values than Huhan-1A on the 3rd DAT (Figure 1A).

The total soluble protein content (Figure 1B) and chlorophyll content (Figure 1H) were down-regulated as a result of oxidative stress. Huhan-1A had higher values for both traits than Huhan-1B on the 3rd DAT (see Figure 1). The H_2_O_2_ content, GPX activity, SOD activity, and CAT activity were all up-regulated via oxidative stress in both Huhan-1A and Huhan-1B (Figure 1). However, the values of these physiological traits of Huhan-1B were higher than those from Huhan-1A on the 3rd DAT. The AOC in Huhan-1A was significantly higher than the AOC of Huhan-1B on the 1st DAT, while it was lower in Huhan-1A than in Huhan-1B on the 3rd DAT (Figure 1D). The final survival ratio of Huhan-1A was higher than the Huhan-1B recovery of the 5th DAT (see Figure 1I).

### Efficiencies of RNA editing of mitochondrial genes in response to oxidative stress

A total of 90 editing sites were detected on all six mitochondrial genes in Huhan-1A and Huhan-1B prior to (0 DAT) and during (1st and 3rd DAT) the oxidative stress (Table 1 and Table S3). DNA sequences of target regions in these mitochondrial genes were identical between Huhan-1A and Huhan-1B, which was particularly apparent at the detected editing sites (Figure S1). Forty-eight sites were found to significantly regulate their respective editing efficiencies in Huhan-1A and/or Huhan-1B when they encountered oxidative stress (Table 1 and Table S3). These were thus defined as stress-responsive sites (SRSs). Surprisingly, these two rice lines shared only five such SRSs (Table 1). Additionally, 62 sites represented significantly different editing efficiencies between Huhan-1A and Huhan-1B at least during one time point (see Table 1 and Table S3). These were defined as differently edited sites (DESs). Interestingly, all SRSs in Huhan-1A increased their editing efficiencies, while most of those in Huhan-1B decreased their editing efficiencies (Table S3). As a result, the degree of differences of editing efficiencies on these responsive sites between Huhan-1A and Huhan-1B decreased on the 1st and 3rd DAT, compared to the differences on 0 DAT (Figure S3). Additionally, most differently edited sites represented lower editing efficiencies in Huhan-1A, except for three sites at *ccmC* (site115, site161, and site169) (Table S3). It was also worth noting that changes of amino acids on most stress-responsive or differentially edited sites changed from polar to hydrophobic (Table 1 and Table S3).

**Table 1:** Statistics of stress-responsive sites (SRS) and differentially edited sites (DES) detected within six mitochondrial genes and their alterations of amino acid properties *via* RNA editing.

**Gene**	**No. of sites**	**Alteration of amino acid property**			**No. of SRS**			**No. of DES**
		**H to P**	**P to H**	**Unchanged**	**Huhan-1A**	**Huhan-1B**	**Shared**	
*atp9*	3	0	1	2	0	0	0	0
*nad2*	5	0	1	4	0	3	0	5
*nad7*	13	1	6	6	1	1	1	0
*nad9*	9	1	5	3	0	7	0	2
*ccmC*	27	3	11	13	4	6	0	22
*ccmB*	33	5	17	11	11	20	4	33
Total	90	10	41	39	16	37	5	62

“*H” and “P” are abbreviations for “hydrophobic” and “polar.”*

A total of three editing sites were detected in *atp9* (Tables S1, S3). Oxidative stress had no detectable impact on RNA editing efficiencies of the individual sites of *atp9* (Table S3). Their editing efficiencies were also not significantly different between Huhan-1A and Huhan-1B on the 0, 1st, and 3rd DAT (Table S3). However, the general editing efficiency of *atp9* in Huhan-1A decreased significantly from 68.3 to 49.7% (Table 2).

**Table 2:** General editing efficiencies (mean ± SE) of mitochondrial genes on the 0, 1st, and 3rd days after treatment (DAT) in Huhan-1A and Huhan-1B.

**Gene**	**No. of sites**	**Editing efficiency in Huhan-1A**			**Editing efficiency in Huhan-1B**		
		**0 DAT**	**1 DAT**	**3 DAT**	**0 DAT**	**1 DAT**	**3 DAT**
*atp9*	3	^*^68.3 ± 1.9b	^*^49.2 ± 1.8a	^*^49.7 ± 6.1a	43.2 ± 6.6	31.6 ± 5.5	30.4 ± 5.0
*nad2*	5	88.8 ± 1.5	89.0 ± 2.2	89.9 ± 2.6	93.5 ± 3.0	86.7 ± 7.5	90.6 ± 2.0
*nad7*	13	93.3 ± 6.4	93.7 ± 6.0	93.9 ± 5.3	91.1 ± 6.0	92.8 ± 6.0	93.5 ± 5.2
*nad9*	9	^*^72.1 ± 1.1b	^*^65.0 ± 1.7a	^*^67.2 ± 1.6a	81.1 ± 0.8b	72.4 ± 1.1a	73.4 ± 2.0a
*ccmC*	27	^*^41.2 ± 5.5	^*^52.3 ± 5.0	^*^51.6 ± 5.0	82.9 ± 1.1	81.2 ± 1.5	81.0 ± 1.2
*ccmB*	33	^*^37.3 ± 4.0	^*^43.0 ± 4.1	^*^47.8 ± 3.4	86.5 ± 3.4	82.6 ± 3.1	83.4 ± 3.0

*General editing efficiency was calculated as the means of editing efficiencies at all editing sites of a mitochondrial gene*.

* *Indicates significant difference (p < 0.05) between Huhan-1A and Huhan-1B obtained via paired t-test. Different letters behind values indicate significant differences (p < 0.05) among time points based on one-way ANOVA (SNK method)*.

Six editing sites were detected at *nad2* (Table 1 and Table S3). Three SRSs of *nad2* (site2814, site2880, and site2910) had decreased editing efficiencies in Huhan-1B in response to the oxidative stress, while no SRS were detected in Huhan-1A. Furthermore, four sites (site2810, site2814, site2880, and site2910) and three sites (site2880, site2910, and site3000) could be defined as DESs of *nad2* between Huhan-1A and Huhan-1B on 0 DAT and the 1st DAT, respectively (Table S3). The general efficiencies of *nad2* were not influenced by stress and were therefore similar between Huhan-1A and Huhan-1B (Table 2).

Thirteen editing sites were detected in *nad7* in total (Table 1 and Table S3). One SRS (site2657), shared by Huhan-1A and Huhan-1B, increased its editing efficiency in response to oxidative stress in both lines (Table S3). No DES were detected between Huhan-1A and Huhan-1B (Table 1 and Table S3). The general efficiency of *nad7* was not influenced by stress and remained similar between Huhan-1A and Huhan-1B (Table 2).

A total of nine editing sites were detected in *nad9*, seven among which could be defined as SRSs in Huhan-1B (Table S3). Two *nad9* sites (site167 and site356) were determined as DESs between Huhan-1A and Huhan-1B on the 1st and 3rd DAT, respectively (Table S3). Additionally, the editing efficiency of *nad9* in Huhan-1A decreased slightly both in Huhan-1A and Huhan-1B in response to oxidative stress (Table 2). The editing efficiency of *nad9* was also significantly lower in Huhan-1A than in Huhan-1B on the 0, 1st, or 3rd DAT (Table 2).

Thirty-three editing sites were detected at *ccmB*, all of which could be defined as DESs between Huhan-1A and Huhan-1B on the 0, 1st, and 3rd DAT (Table 1 and Table S3). Most of these DESs between Huhan-1A and Huhan-1B changed amino acids from polar to hydrophobic (Table S3). In Huhan-1B, seven SRSs had decreased editing efficiencies on the 1st DAT, but then recovered on the 3rd DAT. In Huhan-1B, a further 12 and 1 SRS had decreased and increased editing efficiencies, respectively, in response to oxidative stress (Table S3). In Huhan-1A, 11 SRSs had increased editing efficiencies in response to oxidative stress (Table S3). The general editing efficiency of *ccmC* in Huhan-1A increased by ~10% in Huhan-1A, while it maintained its original level in Huhan-1B under oxidative stress (Table 2). It was also significantly lower in Huhan-1A than in Huhan-1B on the 0, 1st, or 3rd DAT (Table 2).

Twenty-seven editing sites were detected within *ccmC*, 22 among which could be determined as DESs between Huhan-1A and Huhan-1B on the 0, 1st, and/or 3rd DAT (Table 1 and Table S3). Almost one third of all DESs changed amino acids from polar to hydrophobic (Table S3). Editing efficiencies of three sites (site179, site227, and site331) increased, while efficiencies of three sites (site115, site299, and site418) decreased in Huhan-1B in responses to oxidative stress. Furthermore, four sites (site400, site521, site568, and site575) increased their editing efficiencies in Huhan-1A in responses to oxidative stress (Table 1 and Table S3). Similar to *ccmB*, the general editing efficiency of *ccmC* in Huhan-1A increased by ~10% in Huhan-1A, while it maintained the original level in Huhan-1B in response to oxidative stress (Table 2). Furthermore, general editing efficiency of *ccmC* was significantly lower in Huhan-1A than in Huhan-1B on the 0, 1st, or 3rd DAT (Table 2).

### Influence of amino acid substitutions by RNA editing on protein secondary structures of ccmB and ccmC

**Figure 2:**
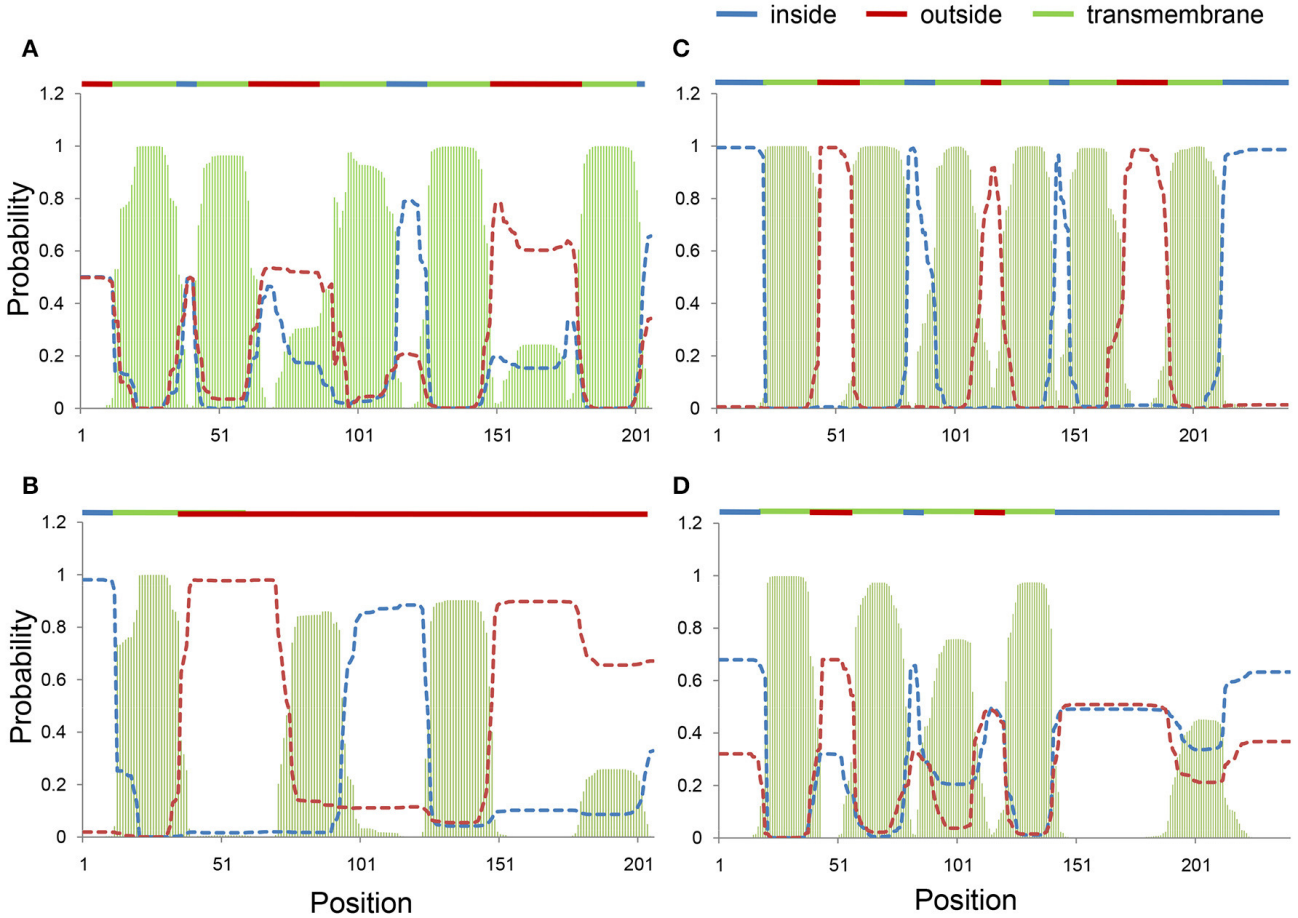
Transmembrane domains in fully-edited (Huhan-1B type) **(A,C)** and non-edited (Huhan-1A type) **(B,D)** forms of ccmB **(A,B)** and ccmC **(C,D)** predicted *via* TMHMM.

Twenty-seven and 26 amino acids were substituted by RNA editing in ccmB and ccmC proteins, respectively (Figure S4). Differences in the amino acid sequences between Huhan-1A and Huhan-1B (resulting from DESs) could lead to large differences in the secondary structures of ccmB and ccmC proteins between both rice lines. The ccmB protein of Huhan-1B type (fully edited) was predicted to possess five transmembrane domains (Figure 2A), while the Huhan-1A type (non-edited) possessed only one predicted transmembrane domain (Figure 2B). Similarly, the ccmC protein of the Huhan-1B type (which was fully edited) was predicted to possess six transmembrane domains (Figure 2C), while that of the Huhan-1A type (which was not edited) possessed only four predicted transmembrane domains (Figure 2D).

### Expressions of six mitochondrial genes and their correlations to RNA editing efficiencies

**Figure 3:**
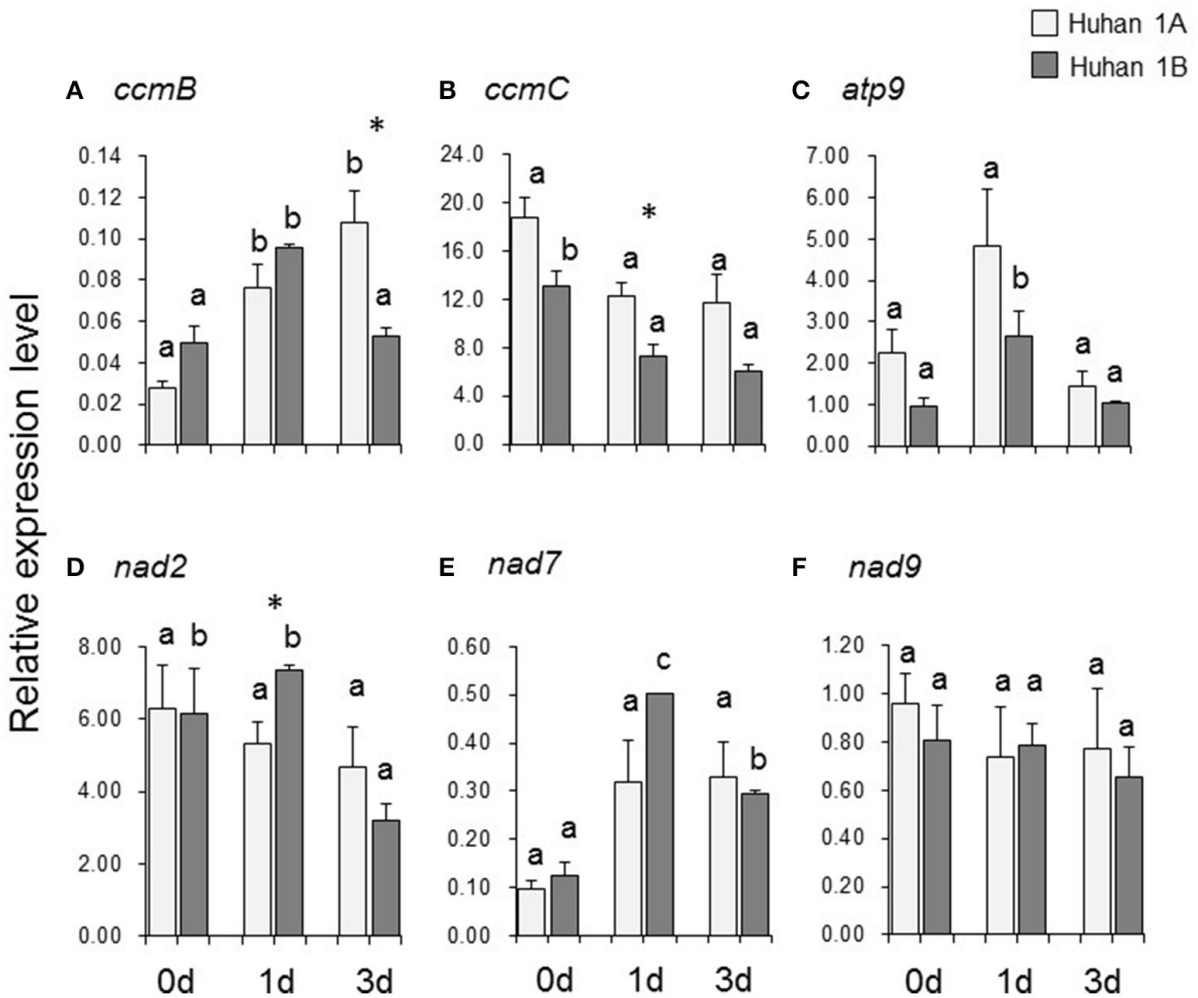
Expressions of six mitochondrial genes (**A**, *ccmB*; **B**, *ccmC*; **C**, *atp9*; **D**, *nad2*; **E**, *nad7*; **F**, *nad9*) in Huhan-1A and Huhan-1B on the 0, 1st, and 3rd DAT. ^*^Indicates significant difference (*p* < 0.05) between Huhan-1A and Huhan-1B obtained *via* independent *t*-test at a time point (*n* = 3). Different letters indicate significant differences (*p* < 0.05) among time points in Huhan-1A or Huhan-1B based on one-way ANOVA (SNK method).

The relative expression level of *ccmB* was significantly up-regulated, both in Huhan-1A and Huhan-1B on the 1st DAT. However, its expression on the 3rd DAT was significantly down-regulated in Huhan-1B compared to the original level on 0 DAT, and therefore, it was significantly lower than that of Huhan-1A (Figure 3A). Oxidative stress down-regulated expressions of *ccmC* (on the 1st DAT) (Figure 3B) and *nad2* (on the 3rd DAT) (Figure 3D) in Huhan-1B, while it did not significantly impact expressions in Huhan-1A. The relative expression levels of *ccmC* (Figure 3B) and *nad2* (Figure 3D) on the 1^st^ DAT differed significantly between the CMS line and its maintainer line. The expressions of *atp9* (Figure 3C) and *nad7* (Figure 3E) were up-regulated on the 1st DAT, but recovered on the 3rd DAT in Huhan-1B. However, oxidative stress had no significant impact on their expressions in Huhan-1A (Figures 3C,E). Oxidative stress did not impact the expression of *nad9* in both Huhan-1A and Huhan-1B (Figure 3F). Its expression was similar in Huhan-1A and Huhan-1B at all three time points (Figure 3F). The editing efficiency was negatively correlated to its expression (*r* = –0.822) at only one site (site331 of *ccmC*).

### Expressions of three PPR genes during oxidative stress and their correlations with RNA editing efficiencies

**Figure 4:**
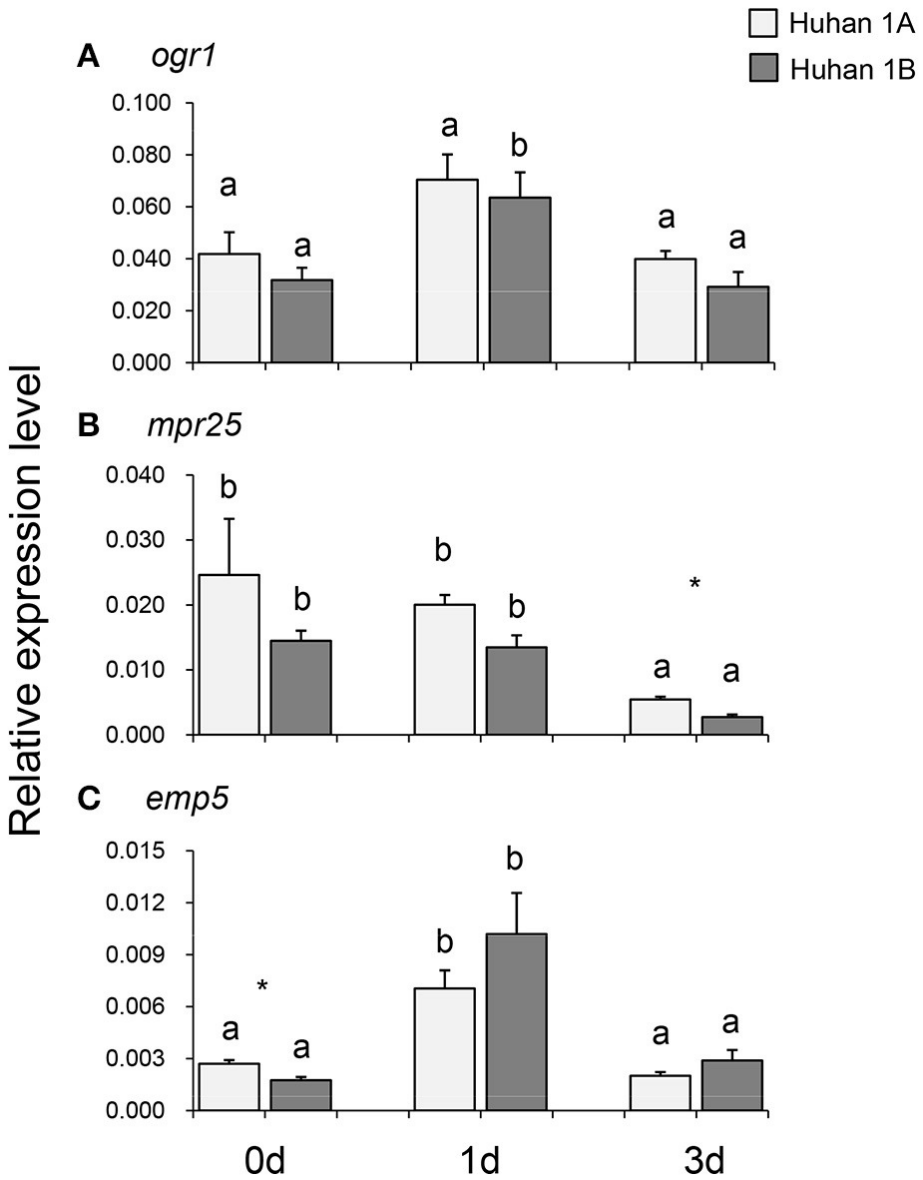
Expressions of three PPR genes (**A**, *ogr1*; **B**, *mpr25*; **C**, *emp5*) in Huhan-1A and Huhan-1B on the 0, 1st, and 3rd DAT. ^*^Indicates significant difference (*p* < 0.05) between Huhan-1A and Huhan-1B obtained *via* independent *t*-test at one time point (*n* = 3). Different letters indicate significant differences (*p* < 0.05) among time points in Huhan-1A or Huhan-1B based on one-way ANOVA (SNK method).

The expression of *org1* remained at the same level on the 0, 1st, and 3rd DAT in Huhan-1A, while in Huhan-1B, it was up-regulated on the 1st DAT and then recovered to the original level. However, its expressions were similar in Huhan-1A and Huhan-1B during all three time points (Figure 4A). The expression of *mpr25* was down-regulated on the 3rd DAT in both Huhan-1A and Huhan-1B. Its expression in Huhan-1A on the 3rd DAT was significantly higher compared to that in Huhan-1B (Figure 4B). The expression of *emp5* was up-regulated on the 1st DAT and then recovered to original levels in both Huhan-1A and Huhan-1B. Its expression was significantly increased in Huhan-1A compared to that in Huhan-1B on 0 DAT (Figure 4C). The expression of rice *mrp25* was negatively correlated (*r* = −0.895) with the editing efficiency on site2657 of *nad7*, while it was positively correlated (*r* = 0.697) with the editing efficiency on site82 of *atp9*. The expression of rice *emp5* was negatively correlated (*r* = −0.693) with the editing efficiency on site3000 of *nad2*.

## Discussion

### WA-CMS and its maintainer line have different responses to oxidative stress

Oxidative stress greatly inhibited the growth of both WA-CMS (Huhan-1A) and its maintainer line (Huhan-1B). However, the maintainer line retained a slight growth during the oxidative stress due to antioxidant enzyme induced activation, resulting in a significantly higher plant height than that of the CMS line on the 3rd DAT. This observation is consistent with previous reports on the performance of rice in 50 mmol H_2_O_2_ treatment between a HL-CMS line (Yuetai A) and its maintainer line (Yuetai B) (Hu et al., 2010; Li et al., 2012). Surprisingly, we detected lower H_2_O_2_ contents on the 3rd DAT under oxidative stress, but a higher survival ratio in the WA-CMS line after re-watering. It is worth noting that the degradation of chlorophyll was stronger in the maintainer line on the 3rd DAT, indicating that Huhan-1B had received greater damage from oxidative stress. This phenomenon has not been reported in previous studies of the HL-CMS line and its maintainer (Hu et al., 2010; Li et al., 2012). Rather, these studies reported an enhanced programmed cell death (PCD) in the HL-CMS line (Li et al., 2012). We suggest a different strategy of stress adaptation between the CMS line and its maintainer line. The maintainer line (whose mitochondrial genome has been domesticated with cultivated rice for thousand years) may prefer a production-first strategy. It maintains a continuous level of growth and increases its antioxidant capacities to scavenge the ROS generated from relatively higher growth under stress. This strategy results in better performance under short and mild stress (Hu et al., 2010; Li et al., 2012). In contrast, the CMS line (whose mitochondrial genome was derived from wild relatives) may prefer a survival-first strategy. With this strategy, the CMS line totally inhibits its growth to reduce ROS production. This stress-adaptation strategy equips the CMS line with a higher survival ratio after long-term and severe stress. Although this hypothesis requires further testing, it could explain the observed performances of wild rice derived CMS lines and their maintainer lines in response to oxidative stress.

### RNA editing on mitochondrial genes differs between WA-CMS and its maintainer line and plays a role in rice adaptation to oxidative stress

The mRNA level of the male sterile gene *WA352* was at the steady state in rice leaf tissues since its transcript did not accumulate in contrast to what has been reported by a previously published study (Luo et al., 2013). As a post-transcriptional mechanism, RNA editing can have a profound impact on mitochondrial functions (Hammani and Giege, 2014), causing further altered performance under normal conditions (Kim et al., 2009; Toda et al., 2012; Liu et al., 2013; Li et al., 2014; Yap et al., 2015). The four mutated PPR genes *slg1* (editing on *nad3*) (Yuan and Liu, 2012), *ahg11* (editing on *nad4*) (Murayama et al., 2012), *slo2* (editing on *nad4L, nad7*, and *mttB*) (Zhu et al., 2014), and *mef11* (editing on *ccmF*_*N*2_) (Sechet et al., 2015) have been shown to be related to stress adaptation via RNA editing on transcripts of many mitochondrial genes. The authors of these studies suggested that deficiencies of editing on specific sites of mitochondria genes could affect the performance of plants under stress conditions. Furthermore, hundreds of PPR genes represent differences in their DNA sequences between Huhan-1A and Huhan-1B. We thus suggest RNA editing on transcripts of mitochondrial genes as the potential cause.

In this study, more than half of all tested sites were in response to oxidative stress since their editing efficiencies altered as the WA-CMS line and/or its maintainer line encountered oxidative stress. Stress-responsive sites (SRSs) in the WA-CMS line were mainly detected in *ccmB* and *ccmC*, all of which increased their editing efficiencies in response to oxidative stress. SRSs in the maintainer line were mainly detected at *nad2, nad9, ccmB*, and *ccmC*. Most of these SRSs slightly decreased their editing efficiencies in response to oxidative stress. It is worth noting that these SRSs were rarely shared between the WA-CMS line and its maintainer line. These results indicate that RNA editing on transcripts of mitochondrial genes responds to oxidative stress and may contribute to their differing performances under stress. In addition, the efficiencies of RNA editing at sites of *atp9* detected in our study are consistent with a previous report using first generation sequencing (Hu et al., 2013), which indicates that the result based on high-throughput sequencing is reliable.

DES between Huhan-1A and Huhan-1B were mainly detected at *ccmB* (all 33 total sites) and *ccmC* (22 DES out of 27 total sites) under both normal and/or stress conditions. Due to different editing efficiencies between Huhan-1A and Huan-1B for both mitochondrial genes, secondary structures of ccmB and ccmC proteins of WA-CMS types (non-edited forms) differed greatly from those of maintainer types (fully-edited forms), which was indicated by the loss of transmembrane domains. Although we did not exactly test whether lower efficiencies of RNA editing on transcripts of *ccmB* and *ccmC* in Huhan-1A could lead to the observed differences in performance of Huhan-1B under stress, loss or decrease of RNA editing on transcripts of numerous other mitochondrial genes in mutants of PPR genes can cause morphological alterations under both normal or stress conditions (Kim et al., 2009; Toda et al., 2012; Liu et al., 2013; Yap et al., 2015). For example, rice *mrp25* is essential in the RNA editing at *nad5* and its mutant exhibited growth inhibition due to reduced photosynthetic capacities (Toda et al., 2012; Yap et al., 2015). Similarly, *ogr1* (Kim et al., 2009), and *emp5* (Liu et al., 2013) play an important role in RNA editing of transcripts of many mitochondrial genes in rice (*ogr1* for *nad2, nad4, ccmC, cox2*, and *cox3*; *emp5* for *nad9, cox2, rpl12*, and *rpl16*). Mutations of *ogr1* and *emp5* have been shown to exert negative influences on plant development and pollen/seed formation (Kim et al., 2009; Liu et al., 2013). Interestingly, both *ccmB* and *ccmC* are associated with the maturation of mitochondrial ETC (Yang et al., 2017) and potentially play important roles in the plant stress-adaptation (Gleason et al., 2011). Evidently, *ccmC* has been reported to be associated with salt stress tolerance in upland cotton (*Gossypium hirsutum* L.) (Zhang et al., 2015). Different forms of the ccmC protein, as well as the ccmB protein, between Huhan-1A and Huhan-1B, could potentially contribute to their differences in performance under oxidative stress.

Furthermore, the rice mitochondrial genome contains dozens of protein-coding genes and hundreds of ORFs with unknown function (Notsu et al., 2002; Asaf et al., 2016). Differences in RNA editing between WA-CMS and its maintainer line can occur on any other transcript of these mitochondrial genes/ORFs. Given the importance of mitochondria for plant adaptation to abiotic stress (Armstrong et al., 2006; Atkin and Macherel, 2009; Millar et al., 2011), we suggest that RNA editing plays an essential role in the plant adaptation to environmental stress.

### PPR genes are likely responsible for differences in stress-responses of RNA editing between the WA-CMS and its maintainer line

Differences in the stress-response of RNA editing efficiency between WA-CMS and its maintainer line should not be closely related to expressions of mitochondrial genes, as revealed by their poor correlations. In contrast, two out of three selected PPR genes showed significant differences in expressions between the WA-CMS line and its maintainer line. They were also activated in response to oxidative stress and a significant number of genes belong to the PPR family (O'toole et al., 2008; Fujii and Small, 2011) and considerable a proportion of these (including *ogr1* and *emp5*) have differences in the DNA sequence between the WA-CMS and its maintainer line (unpublished data). We suggest that many more PPR genes may express differently between the WA-CMS and its maintainer line, similarly to what we observed from *mpr25* and *emp5* in this study. In addition, a PPR gene with a different form (allele) in the WA-CMS line could also profoundly influence its efficiency in RNA editing, rather than its expression level (Verbitskiy et al., 2010). Moreover, we also detected significant correlations between their expression and editing efficiency at many editing sites. All these results indicate that different alleles and expressions of PPR genes should contribute to differences in editing efficiencies between the WA-CMS and its maintainer line. As a result, we can improve the drought-tolerance of a rice variety by regulating RNA editing of mitochondrial genes *via* gene engineering on PPR genes. However, many questions still remain: (1) whether differences in RNA editing between the CMS and its maintainer line are responsible for their differences in mitochondrial genome or whether they are responsible for their differences in PPR genes. (2) How would RNA editing perform under stress in hybrid rice bred from the WA-CMS line × a restore line? Investigating associations among RNA editing, PPR genes, and rice stress adaptation can provide revealing cues for rice breeding of better tolerances to abiotic-stress.

## Conclusion

The WA-CMS line and its maintainer line performed differently in response to oxidative stress. The WA-CMS line completely inhibited its growth under oxidative stress to ensure a higher survival ratio, while the maintainer line maintained a slight growth, which resulted in a lower survival ratio. Responses of RNA editing to oxidative stress were observed at sites of many mitochondrial genes. We also detected large differences in efficiencies of RNA editing between WA-CMS and its maintainer line at transcripts of *ccmB* and *ccmC*, likely leading to significant alterations of their secondary structures. Compared to those in the maintainer line, ccmB and ccmC proteins in the WA-CMS line lost several of their transmembrane domains. As *ccmB* and *ccmC* have been reported to play roles in ETC maturation in maize and act as stress-resistant genes in upland cotton, we suggest that differences in RNA editing at *ccmB* and *ccmC* between the WA-CMS and its maintainer line could potentially lead to different performances under exposure to oxidative stress. However, this requires further experimental validation. In addition, differences in DNA sequences and expressions of PPR genes between WA-CMS and its maintainer line should be the cause of their different RNA editing. Further studies are required to investigate genome-wide associations of differences (expressions and DNA sequences) within PPR genes with differences in RNA editing between the WA-CMS and its maintainer line. The underlying molecular mechanisms of stress-adaptation that can be attributed to RNA editing at *ccmB* and *ccmC* (or any other mitochondrial genes we have not yet studied) should also provide a valuable investigation. Our findings confirmed the important roles played by mitochondria associated with RNA editing in the rice adaptation to oxidative stress. Stress-adaptation associated with RNA editing at mitochondrial genes should also have significance for hybrid rice breeding, which requires further investigation.

## Author contributions

JX and TT conducted most of the experiments and participated in data analyses. JX also participated in the drafting of the manuscript. SY and ZL participated in the experiments. YL, GL, XY, and LL bred and provided the WA-CMS and its maintainer line used for this study. HX designed the experiment, analyzed the data, and drafted the manuscript. LL was involved in the design of the experiment and the drafting of the manuscript. All authors have critically revised this manuscript before submission and agreed to all aspects of the work.

### Conflict of interest statement

The authors declare that the research was conducted in the absence of any commercial or financial relationships that could be construed as a potential conflict of interest.

## Supplementary material

Figure S1 Alignments of mitochondrial gene sequences containing editing sites (in black and red frames) among Nipponbare, Huhan-1A, and Huhan-1B. The site in the red frame was newly detected as it had not been annotated by NCBI.

Figure S2 Plant height measured in Huhan-1A **(A)** and Huhan-1B **(B)** on the 1st and 3rd DAT in stressed (dark gray) and CK (light gray) conditions. ^***^Indicates significant differences (*p* < 0.001) between plant height in the H_2_O_2_ treatment and CK (*n* = 4).

Figure S3 Comparison of degrees of differences [absolute value of (editing efficiency of Huhan-1B manus editing efficiency of Huhan-1A)] at stress-responsive sites among 0, 1, and 3 DAT. ^**^ and ^***^Indicate significance at *p* < 0.01 and *p* < 0.001.

Figure S4 Substitutions of amino acids (in shade) in fully edited and non-edited forms of ccmB and ccmC.
