# Supplementary figures and images for: RNA Editing Responses to Oxidative Stress between a Wild Abortive Type Male-Sterile Line and Its Maintainer Line
Source: Front Plant Sci. 2017 Nov 28;8:2023. doi: 10.3389/fpls.2017.02023 (PMC5712406; doi:10.3389/fpls.2017.02023)

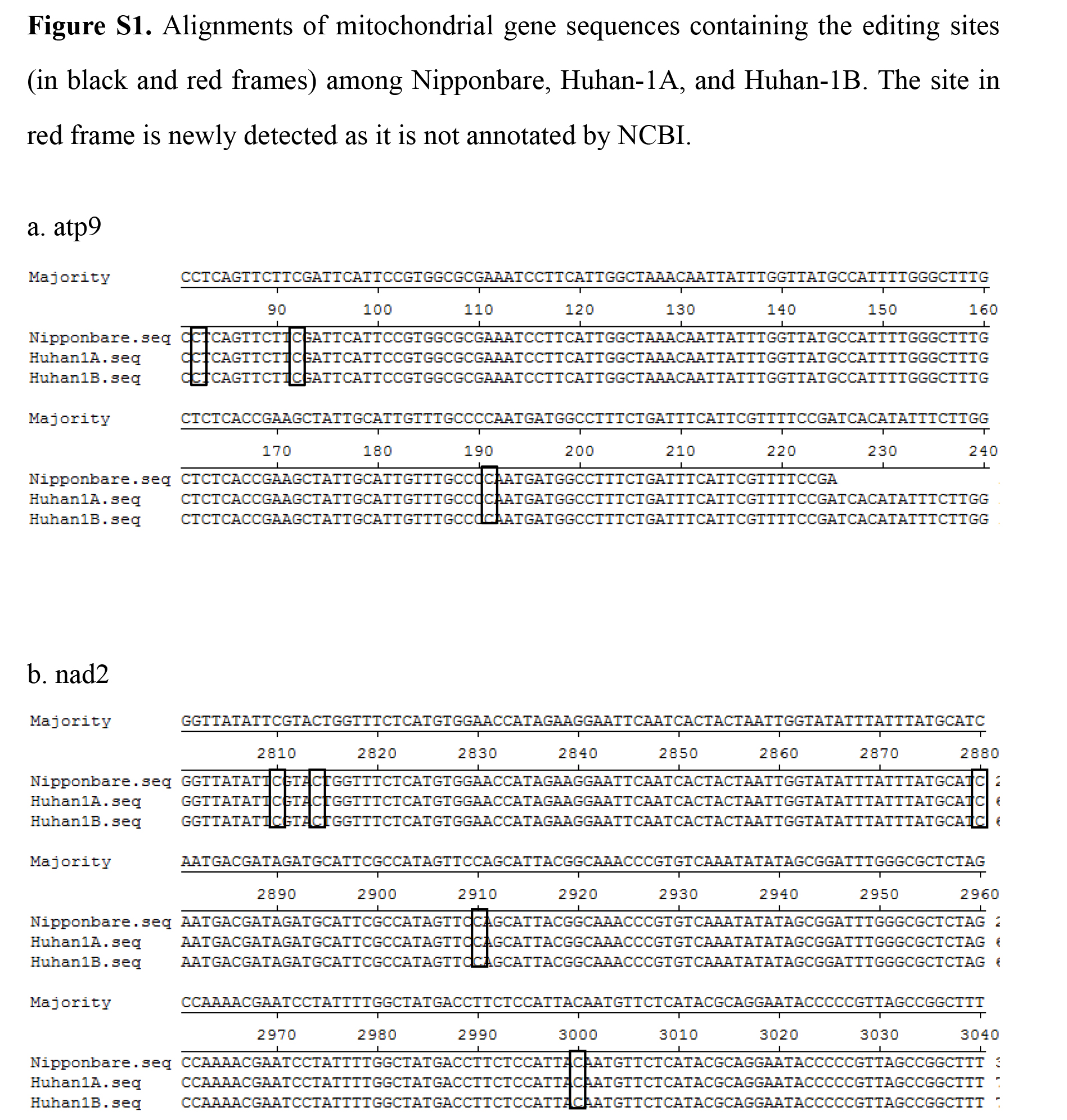

Supplement: Figure S1 — Alignments of mitochondrial gene sequences containing editing sites (in black and red frames) among Nipponbare, Huhan-1A, and Huhan-1B. The site in the red frame was newly detected as it had not been annotated by NCBI. [file Image1.JPEG]

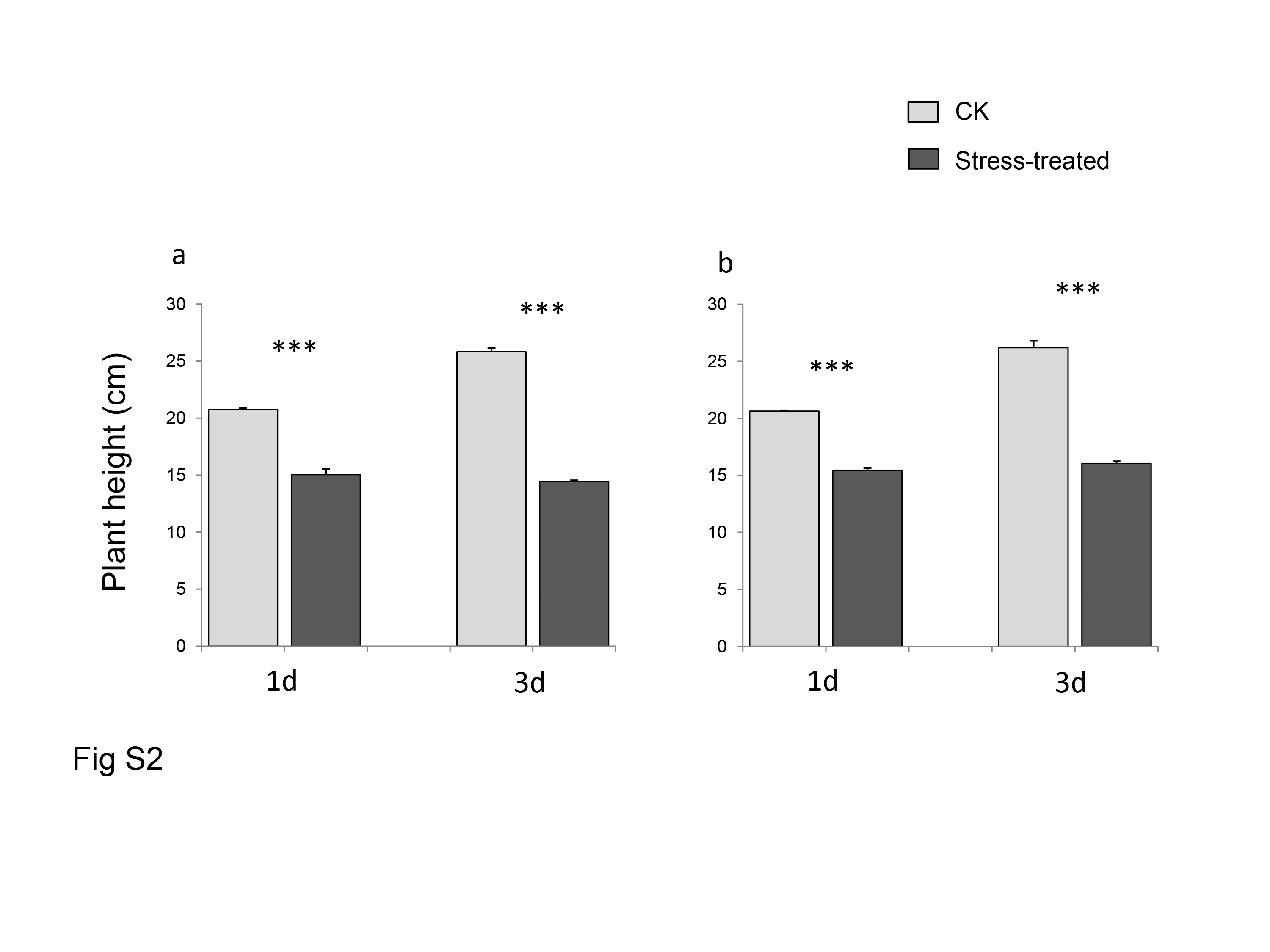

Supplement: Figure S2 — Plant height measured in Huhan-1A (A) and Huhan-1B (B) on the 1st and 3rd DAT in stressed (dark gray) and CK (light gray) conditions. ***Indicates significant differences (p < 0.001) between plant height in the H2O2 treatment and CK (n = 4). [file Image2.JPEG]

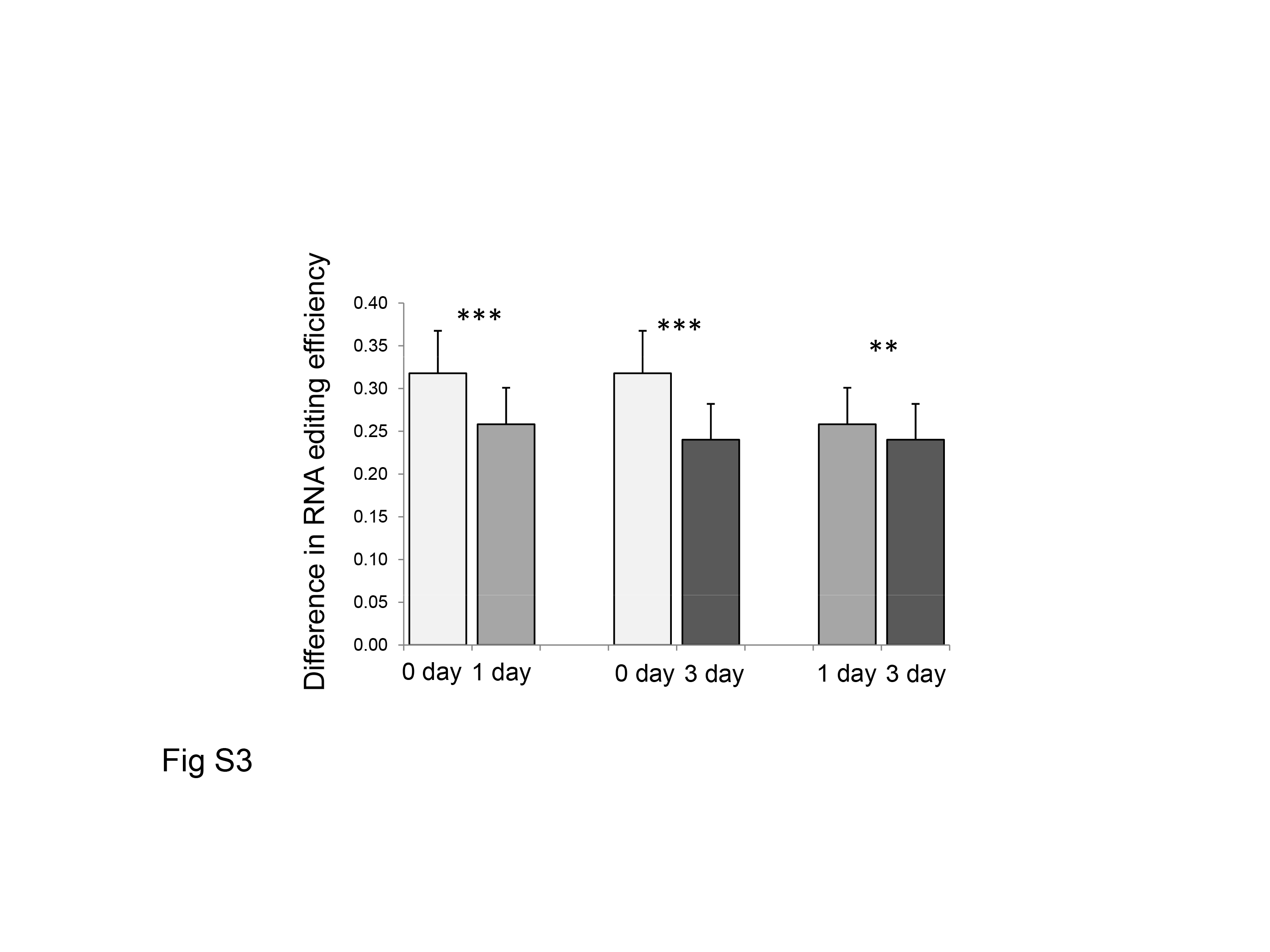

Supplement: Figure S3 — Comparison of degrees of differences [absolute value of (editing efficiency of Huhan-1B manus editing efficiency of Huhan-1A)] at stress-responsive sites among 0, 1, and 3 DAT. ** and ***Indicate significance at p < 0.01 and p < 0.001. [file Image3.JPEG]

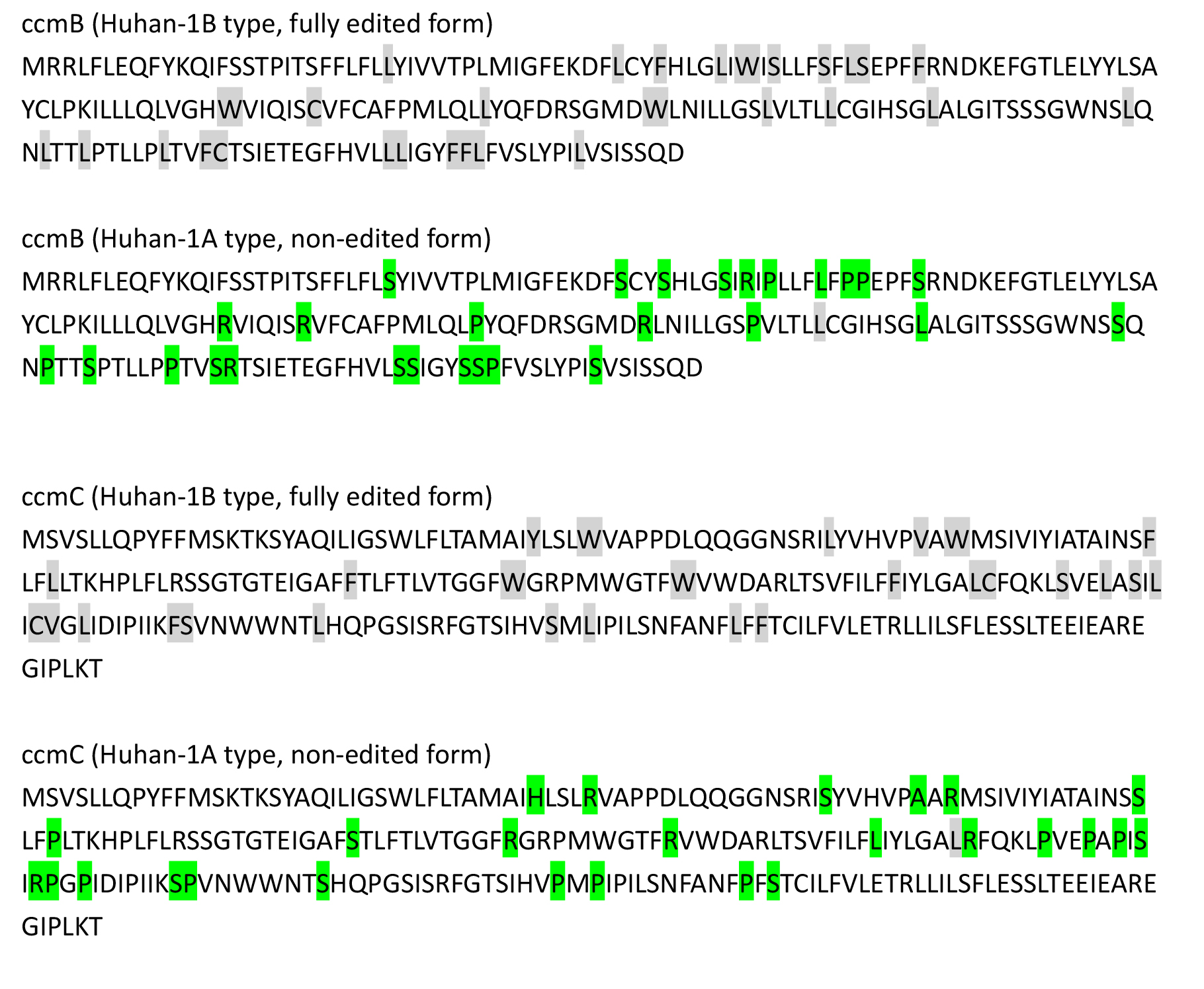

Supplement: Figure S4 — Substitutions of amino acids (in shade) in fully edited and non-edited forms of ccmB and ccmC. [file Image4.JPEG]
