# Supplementary material for: RNA Editing Responses to Oxidative Stress between a Wild Abortive Type Male-Sterile Line and Its Maintainer Line
Source: Front Plant Sci. 2017 Nov 28;8:2023. doi: 10.3389/fpls.2017.02023 (PMC5712406; doi:10.3389/fpls.2017.02023)
Supplement: Supplementary file 5 [file Table1.DOCX]

**Table S1.** Information of SNP and InDel detected between Huhan-1A (28,429Mb clean data, ×71 depth, 352107361 bp covered length) and Huhan-1B (26,869Mb clean data, ×67 depth, 350865093 bp covered length). Nipponbare was used as reference genome (Oryza_sativa.IRGSP-1.0.21.dna.toplevel.fa).

| Category | SNP | | InDel | |
| --- | --- | --- | --- | --- |
|  | Huhan-1A | Huhan-1B | Huhan-1A | Huhan-1B |
| Exonic inframe | 1,180 | 1,071 | 191 | 192 |
| frameshift | 1,506 | 1,399 | 148 | 144 |
| stop_gained | 32 | 28 | 3 | 3 |
| stop_lost | 0 | 0 | 0 | 0 |
| splicing | 19 | 15 | 11 | 10 |
| Intronic | 5,145 | 4,747 | 1,768 | 1,771 |
| coding_sequence_variant | 0 | 0 | 6 | 5 |
| nc_transcript_variant | 1,235 | 1,144 | 395 | 386 |
| UTR | 1,916 | 1,735 | 694 | 685 |
| Upstream/downstream | 17,381 | 16,088 | 5,548 | 5,473 |
| intergenic | 32,677 | 30,108 | 9,118 | 8,937 |
| -- | 6,371 | 11,127 | 387 | 663 |
| Total between Huhan-1A and Huhan-1B | 67,462 | 67,462 | 18,269 | 18,269 |
